# Definition of ideal configuration for femoral neck screw fixation in older people

**DOI:** 10.1038/s41598-019-48258-2

**Published:** 2019-09-09

**Authors:** Jialiang Guo, Weichong Dong, Shiji Qin, Yingze Zhang

**Affiliations:** 1 Department of Orthopaedics, The Third Hospital of Hebei Medical University, Shijiazhuang, P.R. China; 2 Chinese Academy of Engineering, Beijing, P.R. China; 3 Department of pharmacy, The Second Hospital of Hebei Medical University, Shijiazhuang, P.R. China

**Keywords:** Bone, Skeleton

## Abstract

Femoral neck fractures are the most common injuries encountered by older individuals, and they are associated with high mortality and morbidity. Internal fixation to femoral neck fracture with cannulated screws placed with a configuration of an inverted triangle remain a feasible and effective treatment for femoral neck fractures. The objection of this research was to evaluate the femoral neck morphology, especially the shape of the femoral neck in Chinese people to find the optimal screw position and interval between the screws. 96 consecutive normal subjects without any previous proximal femur operation were reviewed. The patients’ information were collected from our database. The minimum of neck canal height (NCHM), neck canal width (NCWM) and inclination angle (AIA and PIA) were measured in different level. There was a significant difference between the AIA and PIA, neck canal height at inferior and superior 1/3 on posterior wall (NCHIP and NCHSP). Although there was a significant difference between the neck canal height at inferior and superior 1/3 on anterior wall (NCHIA and NCHSA, *p* < 0.001), but the changes were small. The shape of the anterior wall was perpendicular to the horizon and almost parallel with the FNA. In contrast, the shape of the posterior wall resembled a reverse question mark. The inverse triangular fixation was in accordance with the morphology of the femoral neck, and triangular fixation had a high risk of perforation, which may lead to nonunion and avascular necrosis. The anterior screw can be inserted easily with the help of a C-ARM, and the posterior screw positioned mildly posterior to the femoral shaft axis is recommended.

## Introduction

Femoral neck fractures are the most prevalent injuries encountered by older individuals, and they are associated with high mortality and morbidity^1,2^. Numerous methods have been proposed for the stabilization of femoral neck fractures^3^. Dynamic hip screws, cannulated screws and so on are commonly used implants^4^. However, in patients who are not fit for arthroplasty, especially young ones, inverted triangle parallel cannulated screws remain a feasible and effective choice^5,6^.

With the characteristics of excellent stability and limited disturbance to the blood supply of femoral head, patients treated with three cannulated screws exhibit good clinical prognosis for the fixation of femoral neck fractures. Although screw fixation is a commonly used treatment for femoral neck fractures, there are very few published studies describing the morphology of the femoral neck^7^. Furthermore, to improve strength and lower rate of nonunion, surgeons usually propose different screw patterns that is close to the cortex and separated. Innovative configurations with four rhombic screws have also been proposed recently. However, different skills may induce many problems especially for young surgeons; incorrectly inserted screws are often found in postoperative examinations and may decrease the stability of the internal fixation.

When a screw is very close to the hip joint surface and still appears to be within the head on radiographs, it is possible that the screw may have perforated the femoral head. Furthermore, it is difficult to determine the exact distance between the anterior and posterior cortex in the same horizontal plane to the femoral neck through 2-dimensional images. A comprehensive understanding of the morphology of the femoral neck is vital to both screw fixation and the clinical results, but the measurement of limited intramedullary space is restricted by technology. The objection of the study was to evaluate the femoral neck morphology, especially the shape of the femoral neck canal in Chinese patients to find the optimal screw insert position and interval between the screws.

## Materials and Methods

### Study subjects

From 2015 to 2016, subjects without any previous proximal femur surgery were reviewed. The patients’ information were collected, retrospectively, and all the methods were performed in accordance with the regulations of our hospital.

The research was conducted, and the subjects were telephoned to ensure that they were willing to participate in 2019. Informed written consent was obtained from all subjects. The inclusion criterion was people without fractures or surgical treatment. The exclusion criteria was people with the presence of a femoral neck fracture or pathological one, history of femoral neck fracture, surgical treatment with open reduction, and data without CT. This research was approved by the ethics committee of our hospital. The registered clinical trial number was NCT03550079.

All the subjects were examined using a Siemens spiral 64-slice multidetector scanner from Germany (Siemens Medical, Freistaat Bayern). The technical parameters were set as follows: 120 kV, 80–110 mAs, pitch 0.9 and an acquisition thickness of 1 mm. The CT scans from iliac spine to the lesser trochanter were reviewed in each subject, and bony deformity was not found. Computed tomography (CT) scans were obtained, and all osseous structures were required to be normal and complete. The normal one side was chosen as our objectives.

### Mimics evaluation

The threshold and region growth processing strategy was used to segment the contours of each proximal femur. In this research, Hounsfield units of 226 (minimum) and 1600 (maximum) were used as the threshold of bone tissue in the femoral neck. The 3D images reconstructed by Mimics (Materialise, Leuven, Belgium) can be rotated freely to measure the length, width and depth on desired planes.

**Figure 1:**
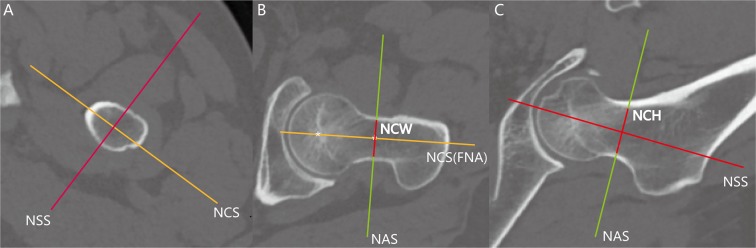
Illustration of the femoral neck axis and projection of the true axial (green line), sagittal (red line), and coronal views (yellow line). (**A**) The neck axial slices are the planes perpendicular to the femoral neck axis. The NAS at the narrowest level is named as NASN (the narrowest slice of femoral neck on NAS). (**B**) The NSS are the planes perpendicular to the NAS and parallel to the FNA. The FNA is defined as a line that connects the center of the femoral neck and head. (**C**) The NCS are planes perpendicular to the NAS, NSS and parallel to the FNA.

**Figure 2:**
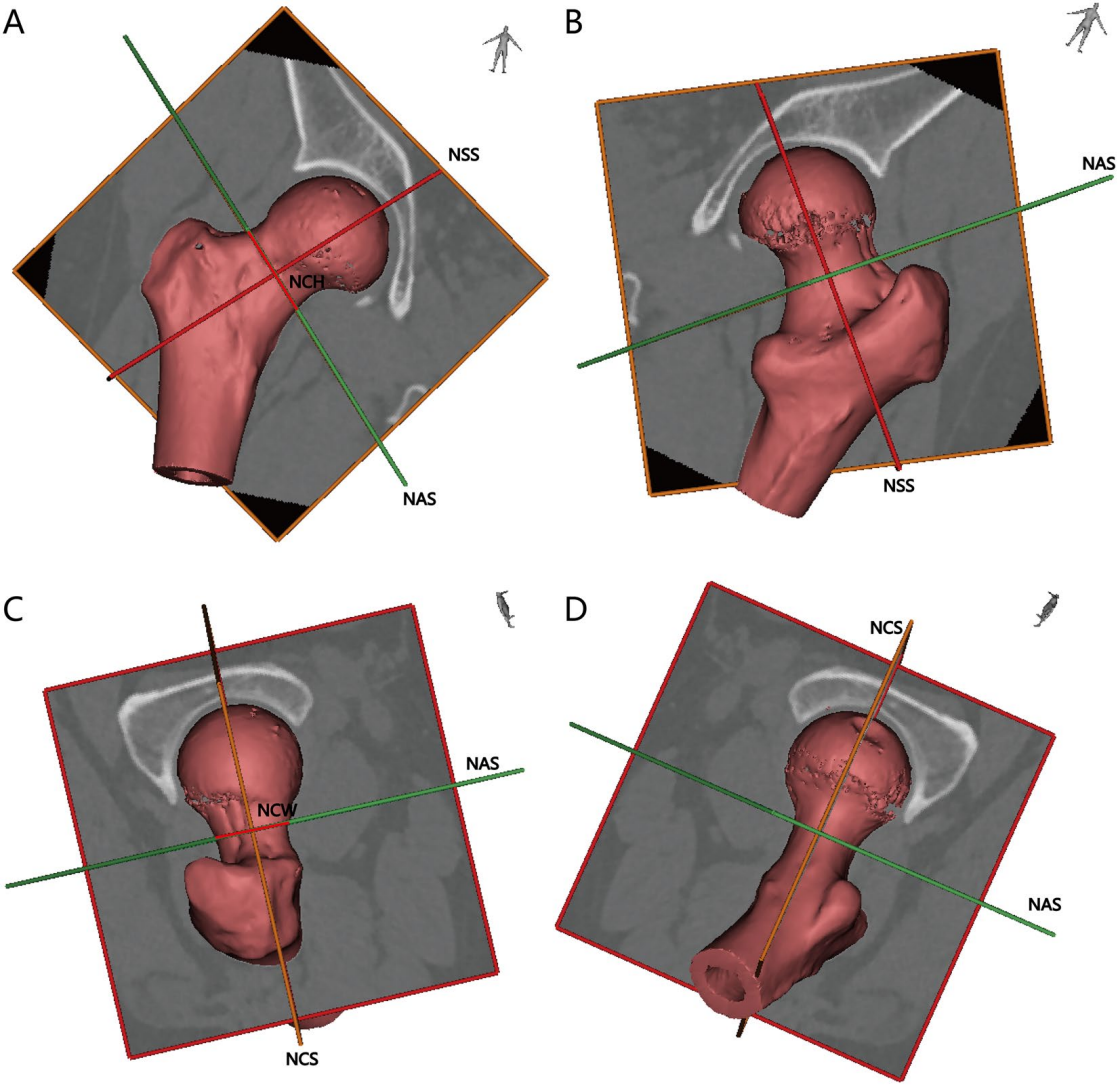
Illustration of 3D images showing the true axial, sagittal, and coronal views. (**A**) The anterior observation of the femoral neck. (**B**) The posterior observation. (**C**) The superior observation. (**D**) The posterior observation.

The following standardized slices were created: the femoral neck axis (FNA) is defined as a line that connects the center of femoral neck and head (Fig. 1). 1. Neck axial slices (NAS) are the planes perpendicular to the FNA. The neck axial slice (NAS) at the narrowest level is named as NASN (the narrowest slice of femoral neck on NAS) (Figs 1A and 2). 2. Neck sagittal slices (NSS) are planes that are perpendicular to the NAS and parallel to the FNA (Figs 1B and 2). 3. Neck coronal slices (NCS) are planes perpendicular to the NAS and NSS and parallel to the FNA (Figs 1C and 2). The relative linear and angular measurements were conducted by the Mimics workstation.

**Figure 3:**
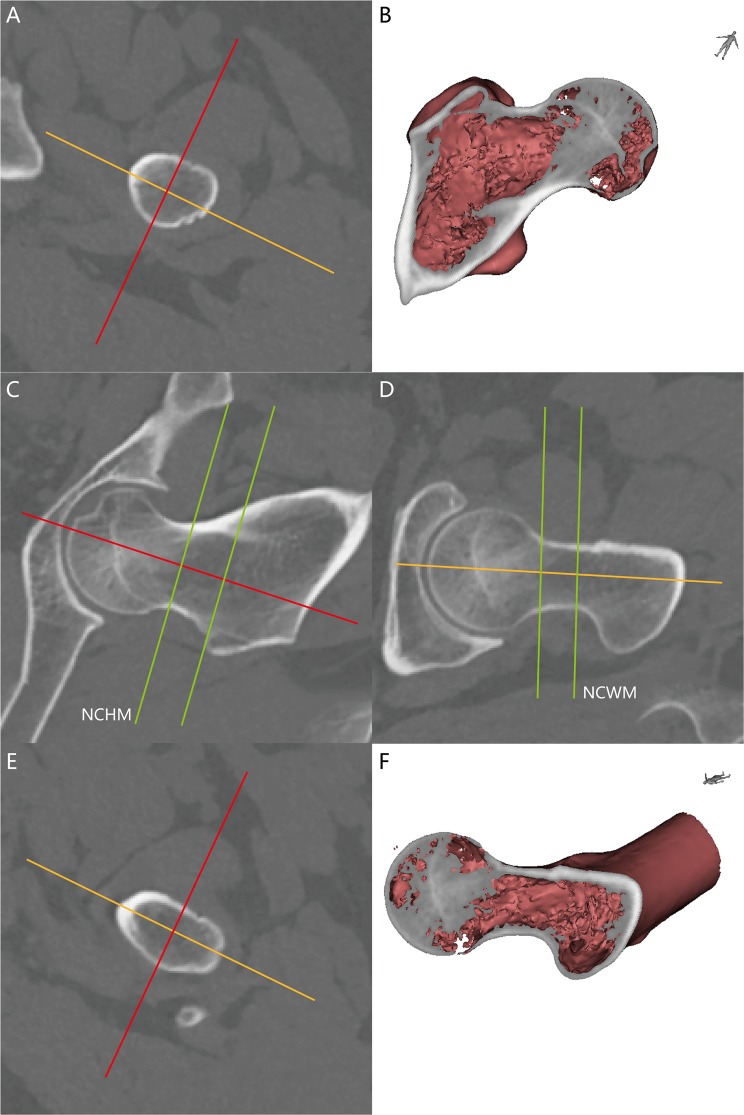
The minimum of neck canal height (NCHM) and neck canal width (NCWM) are measured from the NCS and NSS. The green, red and yellow lines represent the axial, sagittal (the measure direction of NCW) and coronal (the measure direction of NCH) view of the femoral neck. The NSS or NCS were kept to ensure that they passed the center of the femoral neck by visual observation. (**A**) The NAS when the NCHM was observed. (**B**) 3D images exhibit the true coronal view of the NCHM. (**C**) The NCS when the NCHM and NCWM were measured. The not-labeled green line was the axial slice when the NCWM was observed. (**D**) The NSS when the NCH and NCW were measured. The not-labeled green line was the axial slice when the NCHM was observed. We concluded that the NCH and NCW were not on the same slice of the NAS. (**E**) The NAS when the NCWM was observed. (**F**) 3D images exhibit the true sagittal view of the NCWM.

**Figure 4:**
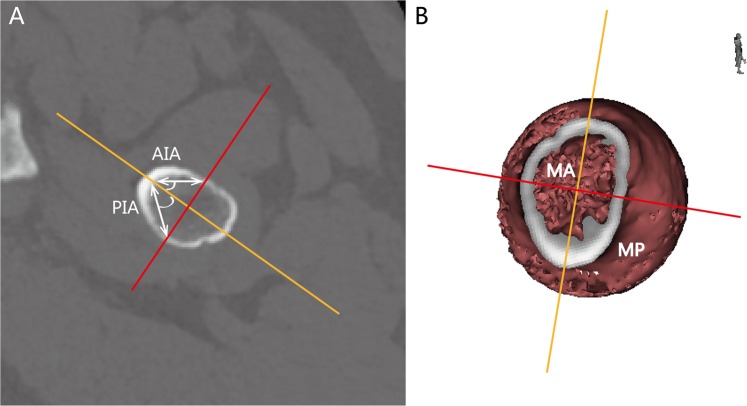
Illustration of the NASN and linear or angular measurement. (**A**) The anterior and posterior wall inclination angles (AIA and PIA) are inclinations to the NCS posteriorly or anteriorly, which pass the PNA on the NASN. (**B**) The minimum perimeter (MP) is the minimum extra cortical length on the NASN. The minimum area (MA) is the total area on the NASN. The NASN of all the measurements can be calculated with the Mimics workstation.

Sagittal or coronal sections were kept to ensure that the center of the femoral neck and head were contained in two slices for measurement accuracy of the parameters on the axial slices (NAS). The neck canal height (NCHM), neck canal width (NCWM) were the minimum length of the femoral neck from the same NSS and NCS. It should be noted that the NCHM and NCWM are not on the same NAS (Fig. 3). The anterior wall inclination angle (AIA) is the inclination to the NCS posteriorly, which passes the PNA on the NASN (Fig. 4). The posterior wall inclination angle (PIA) is the inclination to the NCS anteriorly that passed the PNA on the NASN (Fig. 4). The minimum perimeter (MP) is the minimum extra cortical length on the NASN (Fig. 4). The minimum area (MA) is the total area on the NASN (Fig. 4).

**Figure 5:**
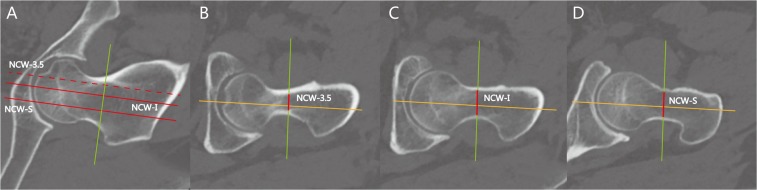
Measurement of the neck canal width for cannulated screws. NCW-3.5 was defined as the parallel line to the FNA with a distance of 3.5 mm from the calcar on the NCS; NCW-I was defined as the distal line parallel to the FNA that trisected the axial line equally; NCW-S was defined as the proximal line parallel to the FNA that trisected the axial line equally. Axial or coronal sections were kept to make the center of the femoral neck and head contained in these two slices to ensure the measurement accuracy of the parameter on sagittal slices. (**A**) The coronal view. (**B**–**D**) The neck canal width on different sagittal views. The red line along the green axis was the identified distance.

Axial or coronal sections were kept to ensure that the center of the femoral neck and head were contained in two slices for measurement accuracy of the parameters on the sagittal slices (NSS). The neck canal width (NCW) is the distance from the anterior wall to the posterior wall along the NAS that passed the center of the femoral neck, demonstrating the space of the screw insertion. Cannulated screws of 6.5 mm are the most commonly used screws for femoral neck fractures in clinic. Therefore, the neck canal width (NCW) was measured at the point of NCW-3.5, NCW-I (inferior 1/3), NCW-S (superior 1/3) on NSS. NCW-3.5 was defined as the parallel line to the FNA with a distance of 3.5 mm from the calcar on NCS; NCW-I was defined as the distal line parallel to the FNA that trisected the axial line equally; NCW-S was defined as the proximal line parallel to the FNA that trisected the axial line equally (Fig. 5).

**Figure 6:**
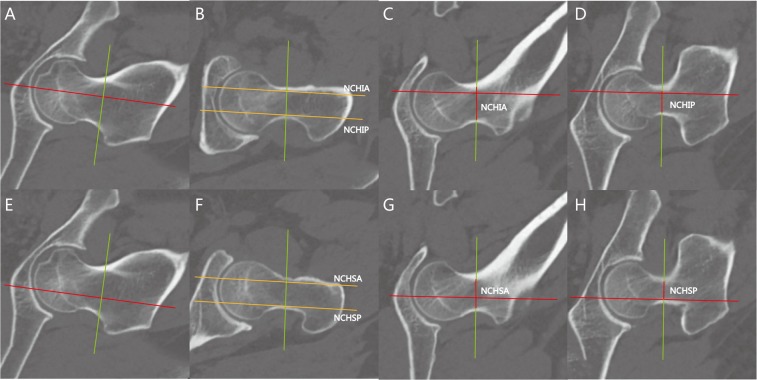
Measurement of the neck canal height for cannulated screws. (**A**) The sagittal slice was determined on NCS (inferior 1/3). (**B**) The sagittal slice. (**C**,**D**) Neck canal height at inferior 1/3 on anterior and posterior wall (NCHIA and NCHIP). (**E**) The sagittal slice was determined on NCS (superior 1/3). (**F**) The sagittal slice. (**G**,**H**) Neck canal height at superior 1/3 on anterior and posterior wall (NCHSA and NCHSP).

Axial sections (NAS) were kept to contain the center of the femoral neck, and the sagittal section (NSS) was moved to the intersection of the superior and middle 1/3, respectively, to measure the neck canal height at the nearest point of the anterior wall or posterior wall (NCHIA, NCHIP, NCHSA, NCHSP) and evaluate the femoral morphology on NCS (Fig. 6).

**Figure 7:**
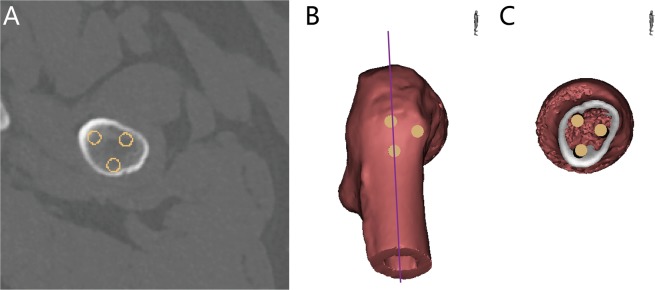
Simulation of the inverse triangular screws. (**A**) The ideal placement of three cannulated screws. (**B**) The 3D images which demonstrated the position of screws. The line represents femoral shaft axis. (**C**) The axial view of the screws originated from (**B**).

A cylinder with a radius of 3.25 mm was used to imitate the screw insertion of the inverse triangular cannulated screws. The optimal entry point of the distal screw was at the level of the lesser trochanter. The ideal entry point of the proximal screws was at the level of upper middle level of the NSS (Fig. 7).

### Statistical analysis

For each parameter, means and standard deviations (Mean ± SD) were calculated. All structures of the femoral neck were measured individually. The statistical analysis was conducted with SPSS 21.0 (Version 21, IBM, Armonk, NY, USA). The Pearson chisquare test and paired *t* test were used to compare the differences between relative data. Fisher’s exact test was performed when the expected frequency was less than 5. P < 0.05 represented there was a statistically significant difference.

## Results

According to the inclusion and exclusion criteria, there were 96 patients (mean age: 53.9 years, range: 45 to 68 years) in our study, including 76 males and 20 females. All the CT scans underwent successful 3D reconstruction. The measurement results (angles, width and height) were illustrated in Table 1. On the NASN, a significant difference was observed between the AIA and PIA (*p < *0.001); the PIA was larger than the AIA. The mean values of the MP and MA were 89.56 ± 9.32 mm and 489.17 ± 112.7 mm^2^, respectively. The minimum of the NCH was close to the junction of femoral neck and head, and the minimum of the NCW was close to the junction of femoral neck and shaft. There was a significant difference between the NCHIA and NCHSA (*p < *0.001), but the changes were small (20.23 ± 3.38 mm vs. 22.53 ± 3.26 mm). Between the NCHIP and NCHSP, a significant difference was also observed (*p* < 0.001), and the changes were obvious (17.73 ± 2.86 mm vs. 13.95 ± 2.67 mm, *p* < 0.001). The shape of the anterior wall was perpendicular to the horizon and almost parallel with the FNA. In contrast, the shape of the posterior wall resembled a reverse question mark. On the NSS, the NCW was increased significantly from inferior 1/3 to superior 1/3 (19.03 ± 2.30 mm vs. 21.42 ± 2.55 mm, *p* < 0.001), and the NCW-3.5 showed that the space for the screw insertion upon the femoral calcar was 13.56 ± 1.6 mm.

**Table 1:** The measurement of femoral neck width and height in different level.

		Normal people	*P* value
**NAS**	AIA (°)	33.7 ± 4.2	
	PIA (°)	38.5 ± 3.4	
			<0.001
	MP (mm)	89.59 ± 9.32	
	MA (mm^2^)	489.17 ± 112.70	
**NCS**	NCWM (mm)	19.76 ± 2.00	
	NCHM (mm)	28.13 ± 3.61	
	NCHIA (mm)	22.53 ± 3.26	
	NCHSA (mm)	21.07 ± 2.95	
			<0.01
	NCHIP (mm)	17.73 ± 2.86	
	NCHSP (mm)	13.95 ± 2.67	
			<0.001
**NCW**	NCW-3.5 (mm)	13.56 ± 1.60	
	NCW-I (mm)	19.03 ± 2.30	
	NCW-S (mm)	21.42 ± 2.55	
			<0.001

AIA, Anterior wall inclination angle; PIA, Posterior wall inclination angle; MP, Minimum Perimeter; MA, Minimum Area; NCWM, Neck canal width minimum; NCHM, Neck canal height minimum; NCHIA, Neck canal height of inferior 1/3 on anterior wall; NCHSA, Neck canal height of superior 1/3 on anterior wall; NCHIP, Neck canal height of inferior 1/3 on posterior wall; NCHSP, Neck canal height of superior 1/3 on posterior wall; NCW-3.5, Neck canal width at 3.5 mm upon femoral calcar; NCW-I, Neck canal width at inferior 1/3; NCW-S, Neck canal width at superior 1/3.

## Discussion

A comprehensive understanding of the morphology of the femoral neck is crucial to screw fixation. Many clinical results had found that the triangle configuration is a risk factor for nonunion in femoral neck fractures; however, no research has focused on morphology to explain the reason. The results exhibited that only one cannulated screw could be placed just upon the femoral calcar. The NCW increased from inferior 1/3 to superior 1/3, and the NCH was decreased mildly in the anterior wall, but distinctly in the posterior wall. From the morphology analysis, the anterior wall was almost parallel to the PNA, and the posterior wall was illustrated as wide space in the upper portion and narrow space in the lower part of the femoral neck. We concluded that the posterior screw should be inserted carefully. The research enriched our understanding of the morphology of the femoral neck, measured the width and height at different levels, defined a safe range of screw insertion, and improved the experience of young clinical orthopedic surgeons.

Cannulated screws are the most widely used for internal fixation in treating femoral neck fractures. Cortical support for the screw position is necessary to obtain stability for the fracture. To achieve cortical support, the screw should be inserted peripherally to close the femoral neck cortex, withstand vertical shear forces and achieve bone union^8,9^. The reduced spread of screws may result in an increased rate of nonunion^10^. To achieve cortical screw support, the morphology of the femoral neck should be known first. In our research, the NCW was measured in three different NSS, and we found that it was increased gradually from the distal to the upper middle intersection of the femoral neck. The upper middle intersection of the femoral neck was considered as the ideal position for proximal screw insertion by most surgeons in the treatment of femoral neck fracture. The mean value (21.42 ± 2.55 mm) shows that a distance of 2–3 screw diameter was allowed on the NCWS. The measurement of NCW-3.5 was to demonstrate the minimum width of the femoral neck needed to achieve cortical support for the distal cannulated screw insertion. The value was 13.56 ± 1.6 mm, and it allows at most two cannulated screws inserted close to each other. When a larger screw (7.3 mm) is used, only one screw can be inserted. From the measurement of the NCW on different NSS, the anatomical rationality of the internal fixation with inverted triangular configuration was proved. The triangular configuration increased the risk of cortical perforation. If the insertion of two distal screws is shown to be safe, the ideal position in the NSS should be elevated almost to inferior 1/3. The reason was that the base side of the triangular configuration should be at least 3 screws in diameter, and the distance was 19.5 mm when 6.5 mm screws were used. The width of the femoral neck at inferior 1/3 was comparable with this demand. However, there were different choices in the clinic depending on patient size; although, achievement of cortical support in triangular screws seemed to be difficult compared with that in inversed triangles.

Furthermore, the NCH at superior 1/3 and inferior 1/3 was also measured in different NSS. The results demonstrated that the NCH increased mildly in the anterior wall of the femoral neck from inferior 1/3 to superior 1/3, and in some patients, no changes were found. In contrast, the NCH increased significantly in the posterior wall. Therefore, the morphology of the posterior femoral neck should be illustrated as a reverse question mark and the anterior femoral neck as a flat surface after a comprehensive measurement of the femoral neck. It seemed that there was more space for screw placement in the posterior wall. However, CT scans in the clinic found posterior screws incorrectly inserted. One reason was that the axis of the femoral neck was not the same as the femoral shaft; the exact position of the screw cannot be figured out accurately on nonstandard lateral views. Another reason was that the insertion of the posterior screw might be affected especially when the anterior screw was not near the anterior wall. When this happened, the large area in the lateral wall of the proximal femur may mislead the insertion of a posterior screw. Therefore, the evaluation of the anterior screw was essential for the posterior one. The screw located near the anterior wall can be inserted easily with the help of a C-arm. The anatomy of the posterior wall could be illustrated as a reverse question mark, and the screw located near the posterior wall should be inserted posteriorly and parallel to the FNA as accurate as possible to avoid wrong insertions. In addition to decreased stability of the internal fixation, the incorrectly inserted posterior screw might injure the deep medial femoral circumflex artery that continues its intracapsular course as the superior retinacular artery. The superior retinacular artery is considered to be the primary blood supply or terminal branch of the superior femoral head 130. Therefore, after the femoral neck calcar screw insertion, the posterior screw can be inserted first to minimize iatrogenic damage during surgical interventions. Furthermore, if the insertion point was low, the risk of in-out-in may also be increased. Due to the anteversion angle, the screws inserted posteriorly were inclined to overlap with the femoral shaft axis through the imitation of the screw trajectory with Mimics. The femoral shaft axis can also help us to improve the accuracy of inserting posterior cannulated screws.

To our knowledge, there are not many reports describing the morphology of the femoral neck. To evaluate the risk of iatrogenic perforation, Zhang found that the screws close to the cortex on both radiographs in the posterosuperior and anteroinferior quadrants should be paid special attention^11^. Nakanishi found the ideal positions for screw insertion; the posterior wall was closer to the femoral axis compared with anterior wall after a study on 50 patients and concluded that the posterior screw should be inserted slightly posterior to the femoral axis^7^. However, apparent deficiencies were observed in this research. The measurement of the cross section was not based on the true axial view of the femoral neck, and the measurement of the linear data was focused on one cross section. In our study, the standardized NAS, NSS and NCS were created, which respected the anatomy of the femoral neck; it was beneficial to measure the relative data on the femoral neck. We noticed that the minimum NCW and NCH were also not in the same NAS. Furthermore, the main problem in Nakanishi’s research was the mistake in identifying the anterior and posterior wall of the femoral neck, which lowers the credibility of the research. However, through the Mimics software, there were significant differences in the NCW and NCH at the anterior wall between superior 1/3 and inferior 1/3; the changes in the NCH at the anterior wall were mild, and it could be considered almost parallel to the FNA. In contrast, the changes in the NCH at the posterior wall between superior 1/3 and inferior 1/3 were noticeable. Therefore, the lateral radiograph of the femoral neck was convincing in reflecting screws positioned near the anterior wall, but ineffective in illustrating the screw position near the posterior wall especially when the images were not standard. Furthermore, there was a significant increase in the PIA compared with the AIA. The correct morphology was necessary to provide comprehensive data supporting the treatment of femoral neck fractures.

To better understand and promote successful internal fixation, the fracture pattern, in addition to the morphology, should also be observed with special attention. Pauwels type 3 femoral neck fractures with high shear loads may lead to nonunions (16% to 59%) and osteonecrosis (11 to 86%) when fixed with internal fixations^12^. Ye reported that cannulated screws combined with a medial buttress plate were used to treat vertical femoral neck fractures, and they improved the fracture union rate^13^. The plate augmentation does not increase the rate of avascular necrosis. In our previous research, we found that cannulated screw augmented with intramedullary nail was another choice in treating vertical ones^4^. After a comprehensive understanding of the anatomy and fracture pattern, it was beneficial to choose optimal internal fixation and decrease complications after femoral neck fractures. The study found a new method of 3-dimensional evaluation of the morphology of the femoral neck. Without the need to collect specimens, the femoral neck canal was evaluated with a specific measurement of 3-dimensional configuration that cannot be evaluated by a 2-dimensional radiographic measurement. The actual axis of the femoral neck, sagittal and coronal views were all set up with the Mimics software. Through 3D reconstruction, the ideal position of the screws can be viewed directly from the 3D images. The traditional measurements on radiographs had different results in various hip rotations, and the results in our research were more accurate.

The limitation of this research was the limited number of patients enrolled. The morphology of young subjects was missing since all the subjects were patients older than 50 years. These results are most beneficial in adults, but should not be extrapolated for fractures in young ones. All the patients enrolled were regular patients without femoral neck fractures, but were still important in guiding the study of the morphology of the proximal femur. In future research, subjects with femoral neck fractures will be included and studied.

In conclusion, this research about femoral neck demonstrated that the inverse triangular fixation was in accordance with the morphology of the femoral neck, and triangular fixation had a high risk of perforation, which may lead to nonunion and avascular necrosis. The anterior screw can be inserted easily with the help of a C-ARM, and the posterior screw positioned mildly posterior to the femoral shaft axis is recommended.

All data measured during the research are included in the supplemental files.

## Supplementary information

original data
