# Supplementary material for: Definition of ideal configuration for femoral neck screw fixation in older people
Source: Sci Rep. 2019 Sep 9;9:12895. doi: 10.1038/s41598-019-48258-2 (PMC6733851; doi:10.1038/s41598-019-48258-2)
Supplement: Supplementary file 1 — original data [file 41598_2019_48258_MOESM1_ESM.pdf]

# Definition of ideal configuration for femoral neck screw fixation in older people

**Jialiang Guo[1]*, Weichong Dong[3]*, Shiji Qin[1], &Yingze Zhang[1,2]**

1 Department of Orthopaedics, the Third Hospital of Hebei Medical University, Shijiazhuang, P R China, 2 Chinese Academy of Engineering, Beijing, P.R. China. 3 Department of pharmacy, the Second Hospital of Hebei Medical University, Shijiazhuang, P R China,*These authors contributed equally to this work.

Correspondence for materials should be addressed to Y.Z.Z. (email: 460706223@qq.com, Work telephone number: 18533112888, Work fax number: 88603000, postcode: 050051

| | | | | | | | | |
|---|---|---|---|---|---|---|---|---|
| 20.38 | 34.42 | 33.17 | 40.04 | 83.88 | 539.34 | 14.36 | 19.58 | 21.53 |
| 21.68 | 33.24 | 33.38 | 37.69 | 80.14 | 644.95 | 14.87 | 22.15 | 22.49 |
| 19.18 | 30.18 | 33.82 | 35.01 | 71.34 | 428.51 | 13.43 | 19.01 | 20.47 |
| 21.52 | 30.71 | 34.03 | 38.44 | 98.57 | 586.67 | 16.63 | 22 | 24.56 |
| 20.01 | 29.17 | 36.2 | 40.29 | 92.77 | 463.35 | 14.44 | 18.37 | 21.24 |
| 19.27 | 25.51 | 35.66 | 38.58 | 81.75 | 520.76 | 12.81 | 20.09 | 19.46 |
| 19.96 | 28.01 | 26.88 | 34.84 | 95.17 | 523 | 14.06 | 19.61 | 22.78 |
| 19.27 | 27.24 | 36.79 | 39.61 | 88.41 | 435.42 | 14.44 | 19.8 | 22.07 |
| 21 | 28.04 | 38.33 | 41.5 | 93.21 | 588.78 | 14.66 | 20.62 | 21.86 |
| 18.54 | 22.6 | 22.57 | 30.56 | 88.19 | 387.64 | 12.13 | 16.63 | 18.96 |
| 20.88 | 26.83 | 30.56 | 42.26 | 98.35 | 516.24 | 15.06 | 18.96 | 20.88 |
| 16.42 | 24.83 | 39.4 | 44.48 | 74.8 | 388.61 | 13.3 | 18.68 | 19.19 |
| 16.634 | 30.55 | 36.6 | 42.37 | 98.02 | 495.45 | 15.71 | 20.18 | 23.56 |
| 18.99 | 28.32 | 32.23 | 39.88 | 103.79 | 592 | 18.68 | 21.86 | 22.49 |
| 16.97 | 21.56 | 26.31 | 30.36 | 73.66 | 213.41 | 11.76 | 14.41 | 17.72 |
| 20.91 | 30.11 | 34.34 | 35.56 | 91.89 | 552.24 | 15.06 | 21.56 | 22.91 |
| 19.19 | 28.14 | 34.32 | 34.32 | 96.39 | 416.07 | 13.19 | 18.45 | 20.84 |
| 22.07 | 28.52 | 40.87 | 36.56 | 90.92 | 536.01 | 13.76 | 19.92 | 22.07 |
| 16.63 | 26.87 | 29.1 | 37.19 | 89.02 | 375.42 | 10.21 | 16.36 | 18.38 |
| 15.68 | 22.41 | 38.55 | 39.16 | 77.42 | 330.73 | 11.14 | 15.95 | 16.96 |
| 21.65 | 31.37 | 34.98 | 41.33 | 97.7 | 602.63 | 13.66 | 20.91 | 25.47 |
| 20.22 | 30.21 | 32.98 | 43.95 | 93.93 | 574.55 | 13.71 | 22.18 | 26.1 |
| 21.53 | 34.31 | 32.56 | 41.02 | 106.2 | 697.54 | 12.39 | 22.07 | 25.44 |
| 16.96 | 24.12 | 35.72 | 36.3 | 84.72 | 330.74 | 10.8 | 14.3 | 16.96 |
| 23.18 | 31.22 | 33.17 | 41.04 | 82.18 | 535.73 | 13.46 | 18.21 | 22.13 |
| 19.68 | 32.54 | 33.38 | 38.69 | 81.24 | 645.65 | 12.37 | 20.35 | 23.03 |
| 18.18 | 32.18 | 33.82 | 37.01 | 70.34 | 429.21 | 14.54 | 21.31 | 22.07 |
| 23.22 | 31.41 | 34.03 | 36.44 | 99.27 | 588.27 | 16.63 | 21.32 | 23.96 |
| 21.01 | 30.17 | 36.2 | 40.29 | 93.97 | 461.55 | 12.94 | 18.87 | 21.24 |
| 20.27 | 26.51 | 35.66 | 38.58 | 80.55 | 521.56 | 10.61 | 17.89 | 18.96 |
| 21.96 | 27.11 | 26.88 | 34.84 | 97.37 | 523.92 | 13.75 | 19.31 | 21.78 |
| 18.27 | 26.34 | 36.79 | 38.51 | 89.11 | 436.62 | 13.98 | 19.27 | 23.07 |
| 20 | 29.14 | 38.33 | 42.5 | 92.21 | 589.48 | 14.66 | 18.12 | 21.86 |
| 19.04 | 21.6 | 22.57 | 34.56 | 89.19 | 386.52 | 12.33 | 16.23 | 19.36 |
| 21.38 | 29.03 | 30.56 | 41.26 | 97.35 | 517.64 | 14.16 | 18.26 | 20.38 |
| 16.92 | 25.43 | 39.4 | 43.48 | 74.28 | 340.91 | 14.3 | 18.68 | 19.59 |
| 16.64 | 31.25 | 36.6 | 41.37 | 96.01 | 493.65 | 16.71 | 20.88 | 23.56 |
| 18.49 | 29.12 | 32.23 | 37.88 | 105.79 | 592.25 | 14.21 | 21.86 | 22.49 |
| 16.47 | 20.16 | 26.31 | 31.36 | 72.96 | 212.31 | 13.35 | 14.91 | 17.72 |
| 21.41 | 31.51 | 34.34 | 36.56 | 92.29 | 550.74 | 13.86 | 19.26 | 22.91 |
| 18.19 | 29.24 | 34.32 | 36.32 | 95.79 | 415.27 | 11.7 | 18.45 | 20.84 |
| 22.17 | 29.12 | 40.87 | 35.56 | 91.52 | 537.41 | 14.76 | 18.72 | 22.07 |
| 18.53 | 25.87 | 29.1 | 35.19 | 87.52 | 377.62 | 12.31 | 17.56 | 18.38 |
| 17.61 | 21.31 | 38.55 | 38.16 | 78.02 | 330.01 | 12.54 | 15.95 | 16.96 |
| 22.75 | 30.27 | 34.98 | 40.33 | 98.7 | 602.43 | 12.12 | 20.91 | 25.47 |
| 23.12 | 31.51 | 32.98 | 42.95 | 95.93 | 571.95 | 12.93 | 22.21 | 24.31 |
| 22.13 | 31.21 | 32.56 | 42.02 | 107.2 | 697.04 | 13.39 | 22.07 | 26.44 |
| 17.32 | 22.02 | 35.72 | 37.3 | 83.72 | 330.04 | 14.85 | 14.3 | 16.96 |
| 22.18 | 32.57 | 33.17 | 39.54 | 80.15 | 542.34 | 13.57 | 18.23 | 20.93 |
| 22.38 | 34.14 | 33.38 | 37.69 | 83.44 | 625.27 | 14.27 | 21.15 | 22.49 |
| 17.18 | 31.28 | 33.82 | 35.01 | 72.34 | 431.61 | 13.93 | 19.01 | 21.07 |
| 21.52 | 29.21 | 34.03 | 38.44 | 99.57 | 585.67 | 15.63 | 20 | 23.06 |
| 22.01 | 30.67 | 36.2 | 48.49 | 91.77 | 467.35 | 16.44 | 18.37 | 22.04 |
| 18.17 | 27.51 | 35.66 | 39.78 | 81.75 | 521.06 | 12.91 | 19.89 | 19.46 |

| | | | | | | | | |
|---|---|---|---|---|---|---|---|---|
| 18.94 | 27.41 | 26.88 | 36.44 | 95.17 | 522.24 | 15.06 | 19.61 | 23.08 |
| 18.27 | 26.94 | 36.79 | 39.61 | 88.41 | 433.92 | 14.74 | 19.8 | 22.07 |
| 20.24 | 26.04 | 38.33 | 41.5 | 93.21 | 586.58 | 14.66 | 20.62 | 22.16 |
| 19.54 | 23.56 | 22.57 | 36.76 | 88.19 | 388.14 | 11.13 | 16.63 | 18.96 |
| 22.88 | 21.83 | 30.56 | 43.16 | 98.35 | 515.74 | 13.06 | 18.96 | 20.88 |
| 18.92 | 25.03 | 39.4 | 45.42 | 74.8 | 389.11 | 13.3 | 18.68 | 20.29 |
| 23.634 | 31.25 | 36.6 | 41.27 | 98.02 | 494.85 | 15.51 | 20.88 | 23.76 |
| 22.59 | 29.32 | 32.23 | 38.98 | 103.79 | 592.37 | 16.68 | 21.86 | 23.49 |
| 15.32 | 21.36 | 26.31 | 31.36 | 73.66 | 212.01 | 11.26 | 14.41 | 17.72 |
| 20.46 | 31.51 | 34.34 | 36.56 | 91.89 | 553.24 | 15.56 | 21.56 | 22.91 |
| 21.14 | 27.04 | 34.32 | 34.32 | 96.39 | 420.07 | 13.89 | 18.45 | 20.84 |
| 21.07 | 27.22 | 40.87 | 35.56 | 90.92 | 534.01 | 13.35 | 19.72 | 21.97 |
| 18.63 | 27.87 | 29.1 | 35.19 | 89.02 | 377.42 | 10.21 | 16.36 | 18.38 |
| 17.28 | 24.51 | 38.55 | 39.16 | 77.42 | 337.73 | 11.34 | 14.9 | 16.06 |
| 23.15 | 31.27 | 34.98 | 41.53 | 97.7 | 600.63 | 13.77 | 20.91 | 24.47 |
| 21.12 | 29.21 | 32.98 | 43.95 | 93.93 | 570.25 | 13.11 | 22.18 | 26.1 |
| 22.73 | 34.81 | 32.56 | 40.52 | 106.2 | 692.59 | 12.09 | 22.07 | 25.44 |
| 18.13 | 20.12 | 35.72 | 36.3 | 84.72 | 330.74 | 11.58 | 15.9 | 18.16 |
| 22.14 | 34.02 | 33.17 | 39.11 | 83.88 | 539.34 | 12.55 | 18.08 | 22.33 |
| 20.18 | 32.14 | 33.38 | 38.69 | 80.14 | 646.95 | 12.87 | 18.15 | 21.49 |
| 21.31 | 31.18 | 33.82 | 36.01 | 71.34 | 430.51 | 12.43 | 19.01 | 21.47 |
| 20.12 | 32.71 | 34.03 | 37.24 | 98.57 | 582.17 | 14.23 | 22 | 24.56 |
| 20.14 | 27.17 | 36.2 | 41.29 | 92.77 | 463.75 | 14.44 | 17.97 | 19.97 |
| 18.37 | 27.21 | 35.66 | 37.28 | 81.75 | 521.76 | 12.81 | 17.89 | 21.06 |
| 19.36 | 27.01 | 26.88 | 32.84 | 95.17 | 523.59 | 15.26 | 17.61 | 21.78 |
| 19.51 | 28.74 | 36.79 | 40.61 | 88.41 | 434.42 | 13.74 | 17.8 | 22.07 |
| 21.31 | 27.04 | 38.33 | 42.5 | 93.21 | 591.78 | 14.96 | 19.52 | 21.06 |
| 19.34 | 23.12 | 22.57 | 32.56 | 88.19 | 385.04 | 13.47 | 16.63 | 19.06 |
| 21.28 | 26.83 | 30.56 | 43.26 | 98.35 | 515.24 | 15.86 | 18.05 | 21.08 |
| 19.72 | 24.83 | 39.4 | 45.48 | 74.8 | 389.01 | 13.83 | 17.98 | 19.95 |
| 21.69 | 30.55 | 36.6 | 41.37 | 98.02 | 494.25 | 15.91 | 21.01 | 23.56 |
| 21.49 | 28.32 | 32.23 | 39.58 | 103.79 | 592.42 | 15.68 | 21.86 | 23.59 |
| 17.57 | 21.56 | 26.31 | 30.86 | 73.66 | 212.01 | 11.06 | 14.41 | 17.92 |
| 21.42 | 30.11 | 34.34 | 35.96 | 91.89 | 551.24 | 13.46 | 21.56 | 22.51 |
| 19.01 | 28.14 | 34.32 | 34.01 | 96.39 | 418.27 | 12.19 | 18.45 | 20.84 |
| 22.34 | 28.52 | 40.87 | 35.26 | 90.92 | 534.24 | 12.76 | 19.72 | 22.07 |
| 19.13 | 26.87 | 29.1 | 38.09 | 89.02 | 370.22 | 11.21 | 16.36 | 18.38 |
| 14.98 | 22.41 | 38.55 | 39.32 | 77.42 | 335.83 | 11.14 | 15.5 | 17.96 |
| 20.15 | 31.37 | 34.98 | 40.33 | 97.7 | 601.03 | 11.36 | 20.91 | 25.47 |
| 23.82 | 30.21 | 32.98 | 44.05 | 94.53 | 576.15 | 12.81 | 22.18 | 25.21 |
| 21.73 | 34.31 | 32.56 | 40.02 | 104.62 | 693.06 | 12.19 | 22.07 | 25.44 |
| 17.36 | 21.12 | 35.72 | 36.53 | 85.82 | 334.14 | 11.28 | 14.3 | 17.06 |

| | |
|---|---|
| 22.14 | 15.87 |
| 20.88 | 19.46 |
| 24.79 | 18.91 |
| 22.18 | 20.88 |
| 20.48 | 17.01 |
| 18.27 | 17.62 |
| 24.83 | 21.87 |
| 21.36 | 15.35 |
| 26.09 | 15.01 |
| 20.26 | 11.76 |
| 24.13 | 18.68 |
| 22.33 | 16.97 |
| 23.06 | 15.23 |
| 23.31 | 16.5 |
| 13.83 | 14.48 |
| 20.31 | 17.62 |
| 21.36 | 17.4 |
| 25.64 | 17.72 |
| 18.45 | 17.72 |
| 21.92 | 16.36 |
| 26.75 | 23.49 |
| 24.45 | 22.41 |
| 30.45 | 22.52 |
| 23.33 | 14.58 |
| 23.26 | 13.87 |
| 20.88 | 19.46 |
| 23.99 | 18.91 |
| 22.18 | 20.08 |
| 20.48 | 18.91 |
| 18.27 | 17.62 |
| 24.83 | 21.87 |
| 22.96 | 16.35 |
| 25.59 | 15.91 |
| 21.26 | 13.26 |
| 23.83 | 18.18 |
| 22.01 | 15.97 |
| 23.06 | 16.23 |
| 23.01 | 15.5 |
| 13.83 | 14.48 |
| 21.01 | 17.62 |
| 21.36 | 17.4 |
| 25.64 | 16.92 |
| 18.45 | 17.22 |
| 20.92 | 16.86 |
| 26.75 | 23.49 |
| 24.45 | 22.41 |
| 20.95 | 23.12 |
| 22.93 | 13.58 |
| 25.24 | 17.23 |
| 20.88 | 19.06 |
| 24.79 | 18.91 |
| 22.18 | 19.88 |
| 20.48 | 17.92 |
| 18.27 | 17.02 |

| | |
|---|---|
| 24.83 | 21.67 |
| 21.36 | 16.15 |
| 26.09 | 14.01 |
| 20.26 | 15.76 |
| 24.13 | 18.68 |
| 22.33 | 16.97 |
| 23.06 | 15.23 |
| 23.31 | 16.5 |
| 13.83 | 14.48 |
| 20.31 | 16.62 |
| 21.36 | 15.24 |
| 25.64 | 17.72 |
| 18.45 | 17.72 |
| 21.92 | 15.36 |
| 26.75 | 23.29 |
| 24.45 | 22.11 |
| 30.45 | 22.22 |
| 23.33 | 15.98 |
| 21.04 | 14.17 |
| 20.88 | 18.26 |
| 24.79 | 18.91 |
| 22.18 | 20.88 |
| 20.48 | 17.91 |
| 18.27 | 17.22 |
| 24.83 | 21.87 |
| 21.36 | 16.15 |
| 26.09 | 15.31 |
| 20.26 | 11.56 |
| 24.13 | 16.68 |
| 22.33 | 17.97 |
| 23.06 | 15.23 |
| 23.31 | 17.21 |
| 13.83 | 14.48 |
| 20.31 | 16.62 |
| 21.36 | 16.4 |
| 25.64 | 18.72 |
| 18.45 | 17.22 |
| 21.92 | 16.76 |
| 26.75 | 23.32 |
| 24.45 | 29.91 |
| 30.45 | 21.62 |
| 23.33 | 17.28 |
